# The impact of predialytic oral protein-based supplements on nutritional status and quality of life in hemodialysis patients: a randomized clinical trial

**DOI:** 10.1186/s12882-025-03999-3

**Published:** 2025-02-26

**Authors:** Mohamed Mamdouh Elsayed, Mohamed Magdy Abdelkader, Amr Mohamed ElKazaz, Iman Ezzat Elgohary

**Affiliations:** 1 Nephrology and Internal Medicine Department, Faculty of Medicine, Alexandria University, Alexandria, Egypt; 2 Internal Medicine Department, El Qabbary General Hospital, Ministry of Health, Alexandria, Egypt; 3 Internal Medicine Department, Faculty of Medicine, Alexandria University, Alkhartoom Square, El Azareeta, Alexandria, 21131 Egypt

**Keywords:** Predialysis, Protein supplementation, Nutrition, Quality of life, Hemodialysis

## Abstract

**Background:**

Administration of oral nutritional supplements (ONS) inside hemodialysis (HD) units before sessions is an increasingly adopted option. Our aim was to assess the effects of predialytic ONS on nutritional status and quality of life (QOL) in HD patients.

**Methods:**

One hundred HD patients were enrolled in this prospective, multicentric randomized clinical trial. Patients were assigned to receive ONS (25 gm protein powder) 1 h prior to the start of the HD session (predialytic) or maintained on their routine nutrition regimen for 3 months.

**Results:**

At study end, supplemented patients showed a significant increase in serum albumin (*p* < 0.001), and a non-significant decrease in the median subjective global assessment (SGA) score. While in the control group, serum albumin remained stable, and the median SGA score increased significantly (*p* < 0.001). Body mass index and anthropometric measures did not differ between both groups. The supplemented patients showed significant improvement in three subscales of the Kidney Disease Quality of Life-36, without a significant change in QOL in control patients. Supplemented patients had significantly higher blood pressure (BP) (*p* = 0.037), lower urea reduction ratio (*p* = 0.020) and Kt/V (*p* = 0.021), higher serum calcium, lower total cholesterol and lower CRP (*p* = 0.047) levels compared to controls. There was no significant difference between groups regarding serum sodium, potassium, or phosphorus or adverse events.

**Conclusions:**

Predialytic ONS administration may contribute to improvements in serum albumin, and QOL. The effects on BP, CRP, and the reduction in dialysis adequacy, should be carefully considered while adopting such strategy.

**Clinical trials registration:**

ClinicalTrials.gov NCT05952570.

**First registration date:**

2/07/2023.

## Background

Malnutrition and protein energy wasting (PEW) frequently affect patients on regular hemodialysis (HD) [1]. Its pathogenesis has been linked to numerous causes including insufficient diet, uremic toxins, inflammation, loss of nutrients during dialysis and comorbid conditions [2]. Recent studies have connected malnutrition to impaired immunity, more predisposition to infection, increased hospitalization rate, lowered quality of life (QOL) and higher mortality [3–5]. Therefore, to prevent these detrimental effects of malnutrition in HD patients, prompt diagnosis and treatment are crucial [6].

There are numerous methods for evaluating nutritional status in HD patients such as subjective global assessment, body mass index (BMI), serum albumin and anthropometric measures which when used in combination gives better nutritional assessment [7, 8]. Adequate protein and energy intake in the diet for HD patients is strongly recommended by the 2020 Kidney Disease Outcomes Quality Initiative (KDOQI) guidelines [9]. Oral nutritional supplements (ONS) are frequently prescribed to HD patients who are unable to obtain enough protein and energy from a normal diet [10]. There is accumulating evidence that the administration of nutritional supplements to dialysis patients is associated with improvements in nutritional status and QOL [11, 12].

There is debate about the ideal time to administer ONS to HD patients [13]. In most of the published studies, ONS was administered during HD sessions (intradialytic) [14, 15], which has been linked to an increased risk of inadequate dialysis and hemodynamic instability [16]. There is an emerging trend to administer ONS prior to the start of the session upon arrival at dialysis units (predialytic) to ensure adherence and avoid the assumed harmful effects of intradialytic supplementation. There are scarce data regarding the effects of predialytic ONS administration. However, few studies have reported encouraging results with its use [17]. In this study, our aim was to study in depth the effects of predialytic ONS administration on nutritional status, QOL, blood pressure (hemodynamic assessment), CRP (inflammatory marker), and dialysis adequacy in HD patients.

## Methods

### Study participants and design

One hundred patients from different dialysis centers in Alexandria were included in this prospective, multicentric, randomized clinical trial. HD patients aged ≥ 18 years and receiving thrice weekly HD sessions (4 h/session, by high flux dialyzers) for 6 months or more were included. Patients were randomly assigned to consume protein-based ONS or maintain their routine nutrition regimen for 3 months via the block randomization method. The protein supplement in the intervention group was Fresubin protein powder (Fresenius Kabi Deutchland, Bad Homburg, Germany) given at a dose of five scoops (25 gm) every HD session for 12 weeks (60 min prior to the start of the session under direct observation from our staff inside HD units to ensure adherence). Fresubin is a fiber free supplement composed mainly of whey protein. Fresubin provides an energy of 18 kcal per 5 gm (1 scoop), with the majority of that energy coming from protein (97% of total energy, 4.4 gm), with minor amounts of lipids (2% of total energy, 0.05 gm), carbohydrates (less than 1% of total energy, less than 0.05 gm) and minerals. Using the sealed closed envelope randomization procedure, allocation concealment was performed, and each patient received a unique identification code. All patients were given dietary recommendations according to KDOQI guidelines with close monitoring during the study. We assessed the dietary intake by using the 3-day food record. All nutritional data of patients were included in their dietary records. We excluded patients with malignancy, liver failure, cognitive impairment, or an allergy to any of the nutritional supplements’ ingredients. We also excluded those who were pregnant, had persistent hyperkalemia or hyperphosphatemia or who had received supplements within the last 12 weeks. The trial was registered on Clinicaltrials.gov (NCT05952570) and was conducted in accordance with the CONSORT 2010 statement.

## Methods

A detailed history was taken from all study participants focusing on demographic data, ESRD etiology, vintage of dialysis, dialysis modality, and comorbid conditions. All patients were physically examined with stress on signs of malnutrition (ex. dry tongue, sunken eyes, muscle wasting, dry skin). Dialysis adequacy evaluation was done using the (second generation) single-pool Kt/V Daugirdas formula [18]. At the end of the session, each participant was questioned about their experience with vomiting, nausea, and intradialytic hypotensive (IDH) episodes. IDH was diagnosed when systolic BP dropped more than 20 mmHg accompanied by symptoms.

### Anthropometric assessment

After the dialysis session, anthropometric measurements including body mass index (BMI), triceps skinfold thickness (TSFT), and mid-arm circumference (MAC) were assessed, and the mean of 3 readings was taken. Weight and height were used to determine BMI (BMI = weight (kg)/height (m^2^), reference value 18.5–24.9 kg/m^2^). A skinfold caliper was used to measure the TSFT. A measuring tape was used to evaluate the MAC.

### Nutritional assessment

Nutritional status was evaluated using the modified subjective global assessment (SGA) scale. The medical history and the physical examination are the two components of the SGA questionnaire. Weight changes, nutritional intake, functional capacity, gastrointestinal issues, and information on diseases and comorbidities are all covered in the medical history section. The physical component focuses on muscle wasting, subcutaneous fat loss, and edema. Patients were classified into three groups based on their total SGA scores: (normal: 7–13), (mild to moderately malnourished: 14–23) and (severely malnourished: 24–35) [19]. Following the dialysis session, the assessment was carried out by the study investigators who had received training from the hospital nutritional specialist on how to calculate the SGA.

### Quality of life assessment using the kidney disease quality of life 36 (KDQOL-36) short form

Each patient’s HRQOL was assessed using the validated Kidney Disease Quality of Life-36 (KDQOL-36): https://www.rand.org/health-care/surveys_tools/kdqol.html [20]. The KDQOL™ -36 is a short version that combines the SF-12 as a core measure with the kidney disease burden, symptoms/problems of kidney disease, and effects of kidney disease (EKD) scales from the KDQOL-SF™v1.3. The five subscales of the KDQOLTM-36 are the Physical Component Summary (PCS), Mental Component Summary (MCS), Burden of Kidney Disease (BKD), Symptoms and Problems of Kidney Disease (SPKD), and EKD. A general assessment of patients’ HRQOL is provided by the first two subscales, while the last three evaluate issues unique to CKD or ESRD patients [18]. For the common questions in the score used, we used an Arabic-translated version of the KDQOL-SF1.3, which was previously established to be valid and reliable to evaluate the HRQOL in ESRD patients, and we translated the remaining questions [21].

The KDQOL-36™ standard scoring program is based on a Microsoft Excel spreadsheet and provides details about the computation process: https://www.rand.org/health-care/surveys_tools/ kdqol.html. Each dimension has a score ranging from 0 to 100, with higher scores indicating better HRQOL [22].

### Laboratory investigations

Pre-dialysis blood samples were obtained and laboratory parameters, including serum albumin (reference value 3.5–5.5 g/dL), creatinine (reference value 0.5–1.3 mg/dL), urea (reference value 18–40 mg/dL), potassium (reference value 3.5–5.0 mEq/L), sodium (reference value 135–145 mEq/L), phosphorus (reference value 2.5 to 4.5 mg/dL), calcium (reference value 8.5–10.2 mg/dL), intact parathormone (reference value 10–70 pg/ml), triglycerides (reference value ˂150 mg/dL), cholesterol (reference value ˂200 mg/dL), C-reactive protein (CRP) (reference value ˂5 mg/L), and complete blood count were measured.

### Statistical analysis

The version 20.0 of the IBM SPSS software package (Armonk, NY: IBM Corp.) was used to analyze the data after data entry to the computer. Numbers and percentages were used to represent categorical data. The chi-square test was used to investigate the association between categorical variables. The Shapiro-Wilk test was used to test for normality in continuous data. The mean and standard deviation were used to express distributed data. Student t-test was used to compare two groups for quantitative variables that are normally distributed, while for comparison between two periods, the Paired t-test was applied. On the other hand, Mann Whitney test was used to compare two groups for abnormally distributed quantitative variables. The results were deemed significant at the 5% level. The Power Analysis and Sample Size Software (PASS 2020) “NCSS, LLC. Kaysville, Utah, USA, ncss.com/software/pass” was used to calculate sample size. To evaluate the effect of predialytic oral protein-based supplements on nutritional status in hemodialysis patients, a minimal total sample size of 100 HD patients (50 per group) was required. A 5% level of significance and an effect size of 50% and 80% power using the chi square-test was taken into consideration.

## Results

### Patients

**Fig. 1:**
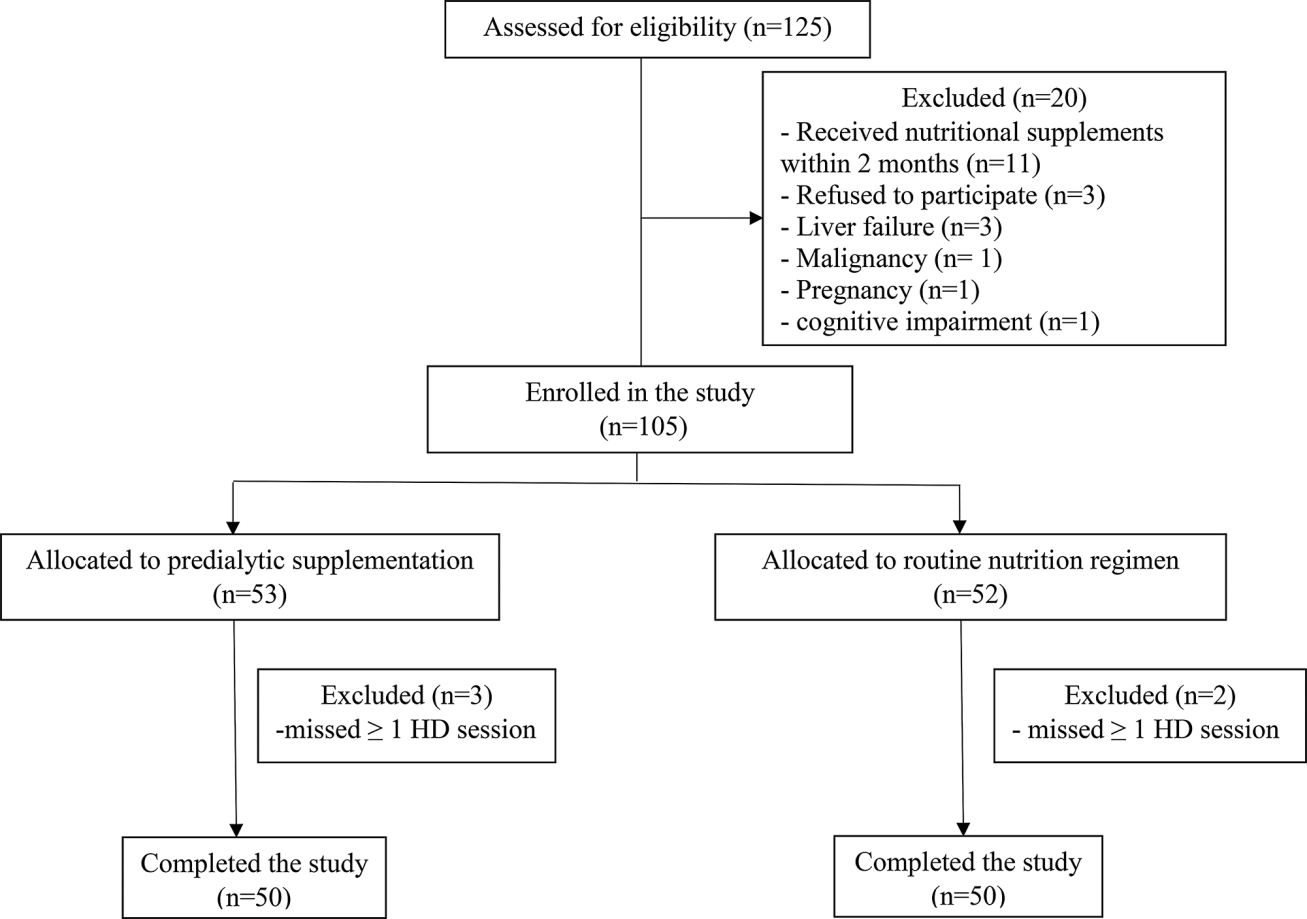
Patient flow chart

One hundred and twenty-five patients were screened. Twenty-two of them were excluded, and three refused to participate. Therefore, a total of 100 patients were enrolled in the study. Following randomization, 50 patients received predialytic ONS, while the other 50 were maintained on their routine diet for 12 weeks (Fig. 1). At baseline, there was no statistically significant difference between both groups regarding age, sex, BMI, cause of ESRD, comorbidities, duration and prescription of HD, vascular access, laboratory parameters, and daily energy and protein intake. The clinical characteristics of the patients are displayed in Table 1.

**Table 1:** Baseline characteristics of the study groups

	Supplemented group (*n* = 50)	Control group (*n* = 50)	*p*
**Age** (years)	52.08 ± 11.37	56.32 ± 13.08	0.087
**Sex**			
Male	21	25	0.422
Female	29	25	
**BMI** (kg/m^2^)	25.36 ± 4.59	26.40 ± 4.35	0.247
**Smoking**	11	12	0.812
**Duration of HD** (years)	3.0 (2.0–5.0)	4.0 (2.0–6.0)	0.374
**Cause of ESRD**			
Hypertension	21	30	0.072
DKD	9	13	0.334
HF	3	1	0.617
Glomerulonephritis (GN)	16	11	0.260
ADPCKD	1	0	1.000
Others	2	3	1.000
**Comorbidities**			
Hypertension	49	44	0.112
Heart failure	11	10	0.806
DM	21	19	0.683
IHD	28	23	0.317
**HD prescription**			
Blood flow rate (ml/min) (QB)	300.0 (300.0–350.0)	320.0 (290.0–320.0)	0.151
Dialysate flow (ml/hr) (QD)	500.0 (500.0–500.0)	500.0 (500.0–500.0)	1.000
UF volume (L/session)	2.86 ± 0.85	2.86 ± 0.72	0.980
**Vascular access**			
AVF	45	37	0.079
Catheter	5	11	
Graft	0	2	
**Total cholesterol** (mg/dl)	182.2 ± 36.01	185.5 ± 41.27	0.675
**Serum triglycerides** (mg/dl)	133.5 (115.0–150.0)	142.0 (124.0–163.0)	0.097
**Hemoglobin** (g/dl)	10.57 ± 1.77	10.06 ± 1.07	0.081
**Serum albumin** (g/dl)	3.82 ± 0.47	3.72 ± 0.45	0.298
**Serum calcium** (mg/dl)	9.40 ± 1.38	8.99 ± 0.95	0.086
**Serum phosphorus** (mg/dl)	5.78 ± 2.87	5.16 ± 1.53	0.182
**Serum PTH** (pg/ml)	451.0 (317.0–800.0)	425.0(177.0–633.0)	0.175
**Serum sodium** (mEq/L)	138.4 ± 4.14	136.9 ± 4.44	0.080
**Serum potassium** (mEq/L)	5.60 ± 0.90	5.36 ± 0.73	0.148
**CRP** (mg/l)	5.30 (4.0–6.50)	6.0 (4.40–9.0)	0.107
**KT/V Daugridas**	1.38 ± 0.25	1.33 ± 0.18	0.256
**Daily energy intake** (kcal/kg/day)	24.29 ± 3.57	25.38 ± 3.32	0.242
**Daily protein intake** (gm/kg/day)	0.89 ± 0.24	0.91 ± 0.21	0.275

*p*: p value for comparing between the two groups

Normally distributed quantitative data were expressed as Mean ± standard deviation (SD) while non-normally distributed quantitative data were expressed as Median with interquartile ranges (IQR), or absolute numbers as appropriate

ADPCKD: autosomal dominant polycystic kidney disease, AVF: arteriovenous fistula, BMI: body mass index, CRP: C reactive protein, DKD: diabetic kidney disease, DM: diabetes mellitus, ESRD: end stage renal disease, HD: hemodialysis, HF: heart failure, IHD: ischemic heart disease, Kt/V: measuring dialysis adequacy, PTH: parathyroid hormone, UF: ultrafiltration

### Nutritional and HRQOL assessment

At baseline, there were no significant differences between the two groups regarding serum albumin, SGA score, BMI or anthropometric measures. At the end of the study, serum albumin showed a significant increase in the supplemented group (*p* < 0.001) but remained stable in the control group (*p* = 0.830), and the difference was significant between the two groups (*p* < 0.001). The median SGA score in the control group increased significantly (*p* < 0.001) but showed a non-significant decrease in the supplemented group (*p* = 0.622). Additionally, at the end of the study, BMI, TSF and MAC did not significantly differ between both groups (*p* values of 0.367, 0.462 and 0.217, respectively), and within each group in comparison to the baseline values (Table 2).

**Table 2:** Nutritional parameters and KDQOL in the study groups

	Supplemented group (*n* = 50)			Control group (*n* = 50)			Comparison bet. gps. at baseline	Comparison bet. gps. at week 12
	Baseline	Week 12	*P*_0_	Baseline	Week 12	*p*_0_	*P*_1_	*P*_2_
**Serum albumin** (g/dl)	3.82 ± 0.47	4.07 ± 0.43	< 0.001*	3.72 ± 0.45	3.71 ± 0.50	0.830	0.298	< 0.001*
**BMI** (kg/m^2^)	25.36 ± 4.59	25.32 ± 4.69	0.653	26.40 ± 4.35	26.16 ± 4.55	0.074	0.247	0.367
**SGA** (points)								
- Normal well nourished	37 (74.0%)	37 (74.0%)		36 (72.0%)	32 (64.0%)			
- Mild to moderate MN	12 (24.0%)	12 (24.0%)		13 (26.0%)	17 (34.0%)		1.000	0.685
- Severe MN	1 (2.0%)	1 (2.0%)		1 (2.0%)	1 (2.0%)			
- Median (IQR)	11.00 (9.0–14.0)	10.50 (10.0–14.0)	0.622	10.50 (9.0–14.0)	12.0 (10.0–15.0)	< 0.001*	0.835	0.067
**Anthropometric measures**								
- Mid arm circumference (cm)	25.28 ± 3.05	25.26 ± 3.06	0.322	26.54 ± 6.15	26.52 ± 6.49	0.799	0.197	0.217
- Triceps skin fold (mm)	21.0 (11.0–22.0)	21.0 (11.0–22.0)	0.655	14.0 (11.0–22.0)	14.0 (11.0–22.0)	0.258	0.550	0.462
**KDQOL subscales**								
- Symptom/ problem list	67.71 (52.08–81.25)	79.17 (62.50–89.58)	< 0.001*	64.58 (45.83–75.0)	66.67 (50.0–77.08)	0.005*	0.207	0.001*
- Effects of kidney disease	50.0 (28.13–65.63)	54.69 (37.50–65.63)	< 0.001*	32.81 (18.75–56.25)	37.50 (21.88–56.25)	0.186	0.056	0.001*
- Burden of kidney disease	18.75 (6.25–31.25)	18.75 (6.25–31.25)	0.257	12.50 (0.0–25.0)	12.50 (6.25–25.0)	0.248	0.057	0.161
- SF-12 physical composite	30.0 (22.95–35.05)	33.36 (23.96–41.54)	0.002*	30.73 (22.58–35.25)	28.74 (21.50–34.71)	0.203	0.948	0.039*
- SF-12 mental composite	39.64 ± 6.81	40.08 ± 5.41	0.655	39.83 ± 6.72	40.08 ± 5.53	0.755	0.891	0.999

Normally distributed quantitative data were expressed as Mean ± standard deviation (SD) while non-normally distributed quantitative data were expressed as Median with interquartile ranges (IQR), or absolute numbers as appropriate

^*t*^*p*_*0*_: p value for comparing between baseline and week 12 in each group

*p*_*1*_: p value for for comparison bet. groups at baseline

*p*_*2*_: p value for for comparison bet. groups at week 12

*: Statistically significant at *p* ≤ 0.05

BMI: body mass index, KDQOL: Kidney Disease Quality of Life, MN: malnutrition, SGA: subjective global assessment

The KDQOL™-36 subscales at baseline did not differ between both groups. At the end of the study, the median scores of the “symptom problem” list, the “EKD” and the SF-12 “physical composite” subscales increased significantly in the supplemented group without a significant change in the control group with a significant difference between both groups with *p* values of 0.001, 0.001 and 0.039, respectively. However, the “Burden of kidney disease” and the SF-12 “mental composite” scores showed no significant difference between the two groups (*p* values of 0.161 and 0.999, respectively), and within each group (Table 2).

### Dialysis adequacy and blood pressure

The URR and KT/V at the end of the study dropped significantly in the supplemented group without a significant change in the control group with a significant difference between both groups (*p* values of 0.020 and 0.021, respectively) (Table 3).

**Table 3:** Dialysis adequacy and blood pressure readings in the study groups

	Supplemented group (*n* = 50)			Control group (*n* = 50)			Comparison bet. gps. at baseline	Comparison bet. gps. at week 12
	Baseline	Week 12	*p*_0_	Baseline	Week 12	*p*_0_	*p*_1_	*p*_2_
**KT/V Daugridas**	1.38 ± 0.25	1.28 ± 0.20	0.002	1.33 ± 0.18	1.37 ± 0.16	0.190	0.256	0.021*
**URR**	69.23 ± 10.68	65.44 ± 8.36	0.006*	67.38 ± 7.36	68.98 ± 6.51	0.127	0.314	0.020*
**MAP** (mmHg)								
- Pre-HD	107.2 ± 11.27	112.3 ± 11.85		102.9 ± 12.98	106.7 ± 14.71		0.086	0.038*
- After 2 h. HD	105.3 ± 14.38	107.1 ± 13.24	< 0.001*	101.1 ± 15.99	101.0 ± 15.72	> 0.05	0.170	0.037*
- After 4 h. HD	93.14 ± 12.39	98.52 ± 14.25		92.32 ± 17.15	92.74 ± 17.20		0.785	0.070

Data were expressed in Mean ± SD

*p*_*0*_: p value for comparing between baseline and week 12 in each group

*p*_*1*_: p value for comparison bet. groups at baseline

*p*_*2*_: p value for comparison bet. groups at week 12

*: Statistically significant at *p* ≤ 0.05

MAP: mean arterial pressure, URR: urea reduction ratio

Regarding blood pressure, at baseline, the mean arterial pressure (MAP) did not significantly differ between the two groups. At study end, the MAP raised significantly in the supplementation group (*p* < 0.001) but not significantly in the control group (> 0.05). In addition, at the end of the study in the supplemented group, the MAP readings (before and after 2 h of HD) were significantly higher than those in the control group, with *p* values of 0.038 and 0.037, respectively. However, at the end of the session, the MAP showed no significant difference between the two groups (*p* = 0.070) (Table 3).

### Laboratory parameters

At the end of the study, patients in the supplementation group had significantly higher serum calcium, lower total cholesterol and lower CRP levels than patients in the control group, with *p* values of 0.017, 0.022 and 0.047, respectively. There was no significant difference regarding triglycerides, sodium, potassium, phosphorus, PTH or hemoglobin between both groups (Table 4).

**Table 4:** Laboratory parameters in the study groups

	Supplemented group (*n* = 50)			Control group (*n* = 50)			Comparison bet. grps at week 12
	Baseline	Week 12	*p*_0_	Baseline	Week 12	*p*_0_	*P*
Total cholesterol (mg/dl)	182.2 ± 36.01	183.1 ± 34.63	0.724	185.5 ± 41.27	200.0 ± 38.08	0.005*	0.022*
Serum triglycerides (mg/dl)	133.5 (115.0–150.0)	136.0 (113.0–155.0)	0.204	142.0 (124.0–163.0)	139.5 (126.0–167.0)	0.969	0.324
Hemoglobin (g/dl)	10.57 ± 1.77	9.88 ± 1.81	< 0.001*	10.06 ± 1.07	10.14 ± 1.42	0.511	0.430
Serum calcium (mg/dl)	9.40 ± 1.38	9.53 ± 1.09	0.531	8.99 ± 0.95	9.03 ± 0.99	0.786	0.017*
Serum phosphorus (mg/dl)	5.78 ± 2.87	5.67 ± 1.86	0.689	5.16 ± 1.53	5.50 ± 1.39	0.138	0.597
Serum PTH (pg/ml)	451.0 (317.0–800.0)	471.0 (276.0–871.0)	0.129	425.0(177.0–633.0)	485.5 (211.0–716.0)	0.035*	0.301
Serum sodium (mEq/L)	138.4 ± 4.14	135.0 ± 3.46	< 0.001*	136.9 ± 4.44	136.2 ± 3.88	0.330	0.100
Serum potassium (mEq/L)	5.60 ± 0.90	5.35 ± 0.94	0.041*	5.36 ± 0.73	5.35 ± 0.91	0.977	0.991
CRP (mg/l)	5.30 (4.0–6.50)	6.0 (5.0–8.0)	0.180	6.0 (4.40–9.0)	7.0 (5.0–10.30)	0.131	0.047*

Normally distributed quantitative data were expressed as Mean ± standard deviation (SD) while non-normally distributed quantitative data were expressed as Median with interquartile ranges (IQR)

*p*_*0*_: p value for comparing between Baseline and Week 12 in each group

*p*: p value for Comparison between groups at Week 12

*: Statistically significant at *p* ≤ 0.05

### Safety & adverse events

Patients in both groups reported having nausea, diarrhea and IHD episodes with no significant difference between both (*p* values of 0.539, 0.143 and 0.459, respectively). No major adverse events were reported in either group (Table 5).

**Table 5:** Adverse events in the study groups

	Supplemented group (*n* = 50)	Control group (*n* = 50)	Comparison bet. groups *p*
Nausea	21	18	0.539
Diarrhea episodes	6.0 ± 5.02	1.50 ± 0.71	0.143
Hypotensive episodes	5.80 ± 4.01	7.08 ± 5.66	0.459

Data were expressed as mean ± standard deviation (SD), or absolute numbers as appropriate

## Discussion

There is increasing debate among nephrologists that intradialytic nutritional supplementation is associated with increased risks of IDH and dialysis inadequacy. Therefore, predialytic ONS has become an interesting option to overcome these concerns, but only a few studies have tested its efficacy. In the present study, predialytic supplementation significantly improved serum albumin and QOL in HD patients compared to patients without supplementation. To our knowledge, we are the largest and among the earliest trials to assess the comprehensive effects of predialytic ONS in HD patients.

To assess the nutritional status of our patients, we utilized multiple tools rather than a single parameter to obtain a better and more precise assessment. In the present work, we used the SGA as recommended by the 2020 Guidelines for Nutrition in CKD published by the NKF/KDOQI [9]. We found that the median SGA score decreased in the supplemented group but increased significantly in the control group without a significant difference between the two groups at the end of the study (*p* = 0.067). Also at study end, percentage of malnourished patients remained constant (26%) in the supplemented group but increased in the control group from 28 to 36%. In addition to the SGA, serum albumin is frequently used to assess nutritional status in combination with other tools [9]. Lower levels of serum albumin are associated with higher hospitalization and mortality rates [23]. We demonstrated a significant improvement in serum albumin only with predialytic ONS. Our findings reflect better nutritional status with the use of predialytic ONS. The consumption of ONS in the dialysis units could explain the better compliance and improvement in our patients. Similar findings were reported by two recent systematic reviews [11, 24], which reported that ONS use in general (not predialytic in particular) may improve nutritional status in dialysis patients.

In addition, we did not find a change in BMI with ONS use. On the other hand, other researchers revealed that ONS significantly increased BMI [11, 24]. Additionally, we demonstrated that the anthropometric measures did not differ between the supplemented and control groups. This is consistent with the findings of Ren et al. [25] in their systematic review. However, other researchers reported positive effects of ONS on anthropometric measures [26]. These contradictory results between different studies including our study might be due to differences in the duration of ONS consumption. Another reason might be the type of the ONS used whether it is protein-based or non-protein based.

The health-related QOL (HRQOL) represents a crucial outcome in HD patients, in whom poor QOL has been liked to malnutrition, depression, increased morbidity and mortality [27]. The KDQOL™-36 is the most common tool for assessing QOL among HD patients [28]. In the present work, we found that predialytic ONS supplementation significantly improved three of the five subscales of the KDQOL™-36, while the control patients did not show a significant change in QOL. The relation between the predialytic administration of ONS (in particular) and QOL has not been previously tested. Additionally, ONS (given during HD or on non-dialysis days) improved QOL, as reported by other studies [12, 24]. The observed positive effects of ONS on QOL might be due to the improvements in serum albumin and inflammatory markers like CRP.

We revealed in the present study that the MAP values were significantly higher among patients in the supplemented group. This association is important to test because intradialytic nutrition is frequently associated with hemodynamic instability during HD, which limits its use by some nephrologists [16]. Our findings would encourage nephrologists to prescribe predialytic ONS without an exaggerated fear of IDH. This is important because IDH is associated with higher morbidity and mortality. In their crossover study, Rao et al. [17] reported better BP control with predialytic nutrition than with intradialytic supplementation. Also, we found that dialysis adequacy parameters (KT/V, URR) were significantly higher in the control group compared to the supplemented group (in which KT/V and URR decreased but still remained above the target value). On the other hand, other researchers [17, 29] revealed no change in dialysis adequacy with predialytic nutrition. The decrease in dialysis adequacy might be due to the increase in urea generation associated with protein intake or the redistribution of blood in the splanchnic area following feeding.

No previous study has reported the effect of predialytic ONS on laboratory parameters. We found that patients in the supplementation group had lower CRP, lower cholesterol and higher calcium levels compared to controls. However, triglycerides, sodium, potassium, phosphorus, PTH and hemoglobin did not differ between the two groups. Published systematic reviews on ONS use reported no effect on serum sodium, potassium, phosphorus or CRP [11, 24]. We assume that the main determinant of any effect on different electrolytes would be the ONS composition itself (micronutrient concentrations) rather than timing of supplementation.

We reported diarrhea, nausea and IDH episodes among both groups without a significant difference. Other studies reported no difference in adverse events between predialytic and intradialytic nutrition [17].

Our study’s strengths include being the largest trial to assess the effects of the predialytic administration of ONS. Moreover, we assessed multiple aspects, including nutritional parameters, QOL, laboratory parameters, hemodynamics and safety. Another strength point was the administration of the same ONS to all patients (not a meal). However, the present study has some limitations. The follow-up duration (12 weeks) might not be enough, and some findings like SGA, BMI, and anthropometric measures might need longer durations to reveal a change (> 6 months). Additionally, patients in the control group were maintained on their routine diet, which varied between patients despite close monitoring by the study nutritionist. Also, the enrollment of more patients from different HD centers would have added more strength to our results. In addition, the baseline clinical characteristics of the study population, such as CRP, Hb, and albumin, are not balanced between the intervention and control groups. Therefore, the differences observed in these parameters at the end of the treatment should be interpreted with caution.

## Conclusion

The findings of this study suggest that administering ONS in dialysis units before sessions (predialytic) may contribute to improvements in serum albumin levels and quality of life in dialysis patients. However, the potential effects on blood pressure and inflammatory markers, such as CRP, as well as the observed reduction in dialysis adequacy, highlight the need for careful consideration when implementing such interventions. Future large-scale randomized clinical trials with extended follow-up periods are necessary to confirm these observations and further explore the long-term impact of predialytic ONS supplementation.

## Data Availability

Upon reasonable request, the data analysed during this study may be obtained from the corresponding author.

## Abbreviations

ONS: Oral nutritional supplement
SGA: Subjective global assessment
HD: Hemodialysis
QOL: Quality of life
